# Botulinum Toxin for the Treatment of Chronic Notalgia Paresthetica: A Case Report

**DOI:** 10.7759/cureus.96000

**Published:** 2025-11-03

**Authors:** Brian A Moreno, Juan Rodriguez-Puerto, Kristin Haushalter

**Affiliations:** 1 Dermatology, Lake Erie College of Osteopathic Medicine, Bradenton, USA; 2 Dermatology, University of Miami, Miami, USA; 3 Dermatology, Derm360, Miami, USA

**Keywords:** adult dermatology, clinical dermatology, #dermatology, dermatology care, dermatology case report, dermatology consult, dermatology outpatients, general dermatology, inpatient dermatology consults, medical dermatology

## Abstract

Notalgia paresthetica (NP) is a chronic sensory neuropathy characterized by localized pruritus and dysesthesia of the upper back, typically resulting from thoracic dorsal rami entrapment. Treatment is often challenging, with many patients experiencing only partial relief from topical corticosteroids, antihistamines, or intralesional injections. We present the case of a middle-aged female with treatment-resistant NP who failed multiple therapies, including topical and intralesional triamcinolone. After experiencing persistent daily discomfort affecting her quality of life, she was treated with targeted intramuscular onabotulinumtoxinA (Botox) injections to the symptomatic area. The patient reported substantial improvement in symptoms following the second round of injections. This case highlights the potential role of botulinum toxin as an effective therapeutic option in managing refractory NP, particularly in patients who do not respond to standard therapies.

## Introduction

Notalgia paresthetica (NP) is a chronic, localized neuropathic condition characterized by pruritus, paresthesia, or dysesthesia of the upper back, most commonly affecting the medial scapular region. The underlying pathophysiology is believed to involve entrapment or irritation of the posterior rami of the thoracic spinal nerves, particularly T2 to T6, as they traverse through the paraspinal musculature to innervate the skin. This results in altered sensory nerve signaling without an identifiable primary dermatologic lesion, although secondary skin changes such as hyperpigmentation or lichenification may develop from chronic scratching or rubbing [1,2]. These chronic mechanical stimuli can perpetuate the neuropathic cycle by inducing epidermal hyperplasia and local inflammatory mediator release, which in turn exacerbate peripheral nerve sensitization and itch perception. Epidemiologic studies estimate that NP affects up to 10% of the general population, occurring most frequently in middle‑aged and older women [1].

It tends to occur more frequently in middle-aged and older women and has been associated with degenerative spinal changes, such as osteoarthritis or disc herniation, further supporting a neuropathic etiology [1,2]. Diagnostic imaging may reveal spinal pathology in some patients, although this is not universally present. The condition can be persistent and distressing, with a significant impact on quality of life, especially when symptoms become chronic.

Despite being relatively common, NP remains underrecognized and frequently misdiagnosed. Management is often challenging due to the limited efficacy of conventional therapies, which typically include topical corticosteroids, capsaicin, antihistamines, gabapentinoids, or physical therapy [1,2]. Intralesional corticosteroids may offer transient relief but often fail to provide sustained benefit, particularly in chronic or severe cases. As a result, patients may experience long-term discomfort and diminished quality of life. Given its chronicity and the limited durability of standard treatments, exploring novel neuromodulatory options such as botulinum toxin is clinically meaningful, particularly for refractory cases like the one described herein.

Botulinum toxin type A (BoNT-A) has emerged as a promising off-label treatment for neuropathic conditions due to its ability to inhibit peripheral release of neurotransmitters such as substance P, glutamate, and calcitonin gene-related peptide, thereby reducing nociceptive transmission and neurogenic inflammation [3,4]. Meta-analyses and systematic reviews have demonstrated the efficacy of BoNT-A in treating a range of peripheral neuropathic pain syndromes, including postherpetic neuralgia, diabetic neuropathy, and cervical radiculopathy [5,6].

Herein, we present a case of treatment-resistant notalgia paresthetica in a 63-year-old female that demonstrated substantial clinical improvement following targeted intramuscular onabotulinumtoxinA injections. This case supports the growing evidence base for botulinum toxin as a neuromodulatory agent in managing refractory neuropathic pruritus.

## Case presentation

A middle-aged female with a past medical history of migraines and hypothyroidism presented to the dermatology clinic with a one-year history of persistent, localized pruritus and discomfort on the right upper back. She denied any associated rash, but described the sensation as a deep, burning itch that was refractory to over-the-counter emollients and antihistamines. The pruritus interfered with her daily activities and sleep quality.

**Figure 1:**
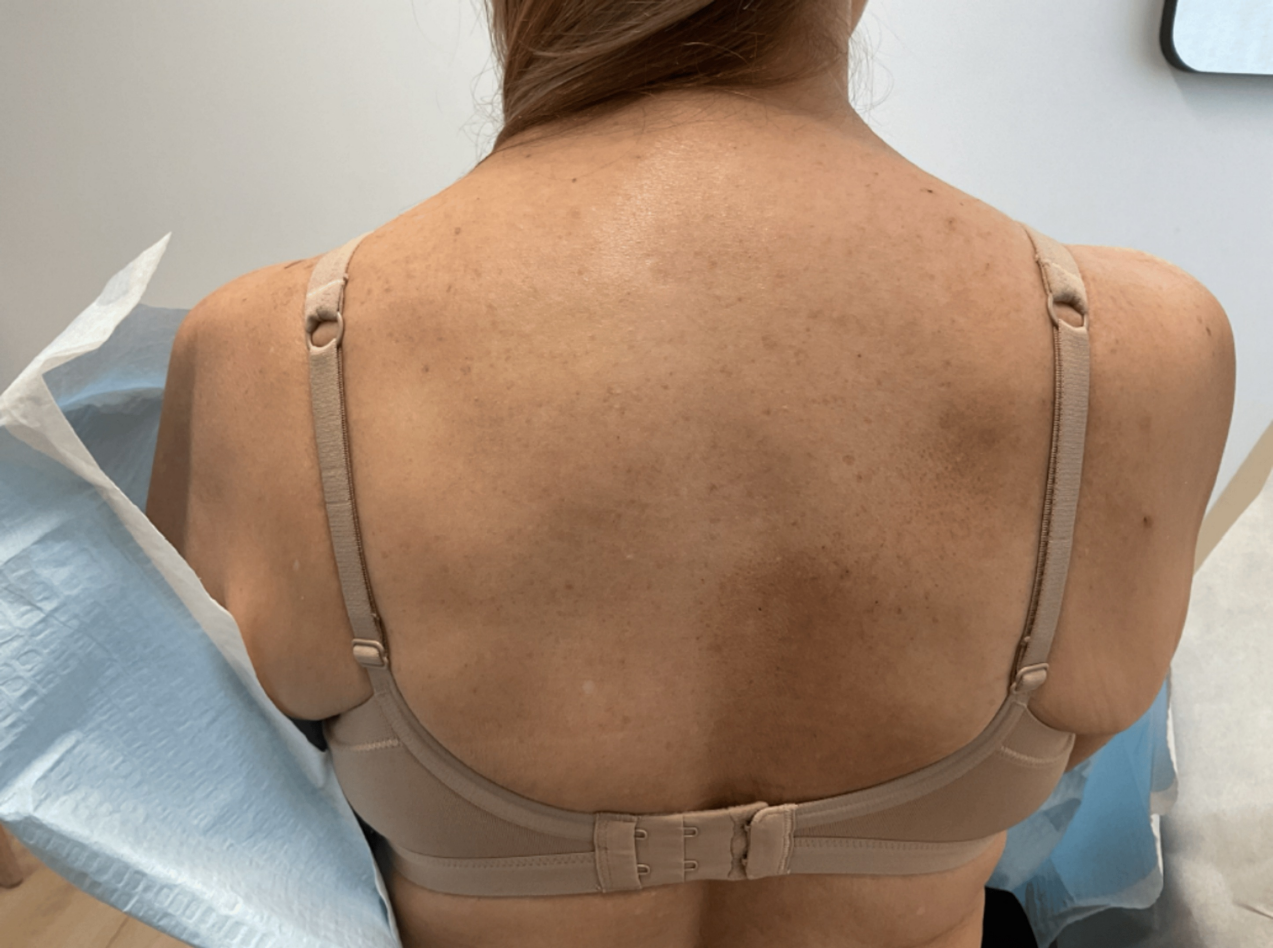
The patient's image at the presentation Post‑inflammatory hyperpigmentation medial to the right scapula, consistent with secondary skin changes from chronic scratching in notalgia paresthetica.

On physical examination, there were no primary dermatologic lesions, but mild post-inflammatory hyperpigmentation was noted in the area medial to the right scapula (Figure 1). The skin was otherwise intact, and there were no signs of infection, lichenification, or excoriation. Based on the clinical presentation, a diagnosis of notalgia paresthetica was made. Differential considerations included contact dermatitis, post‑herpetic neuralgia, and brachioradial pruritus; however, the absence of a primary rash, dermatomal vesicles, or radiating arm pain helped exclude these entities. Spinal imaging was not performed as the clinical findings were consistent with NP, and no neurologic deficits or trauma history were present.

Initial management included a trial of topical triamcinolone acetonide 0.1% ointment applied nightly to the affected area for four weeks. The patient was also instructed to perform daily stretching exercises. At follow-up three months later, she reported minimal improvement. An intralesional injection of triamcinolone (2.5 mg/cc, 0.2 cc total) was administered to the site of maximal pruritus. However, her symptoms persisted.

**Figure 2:**
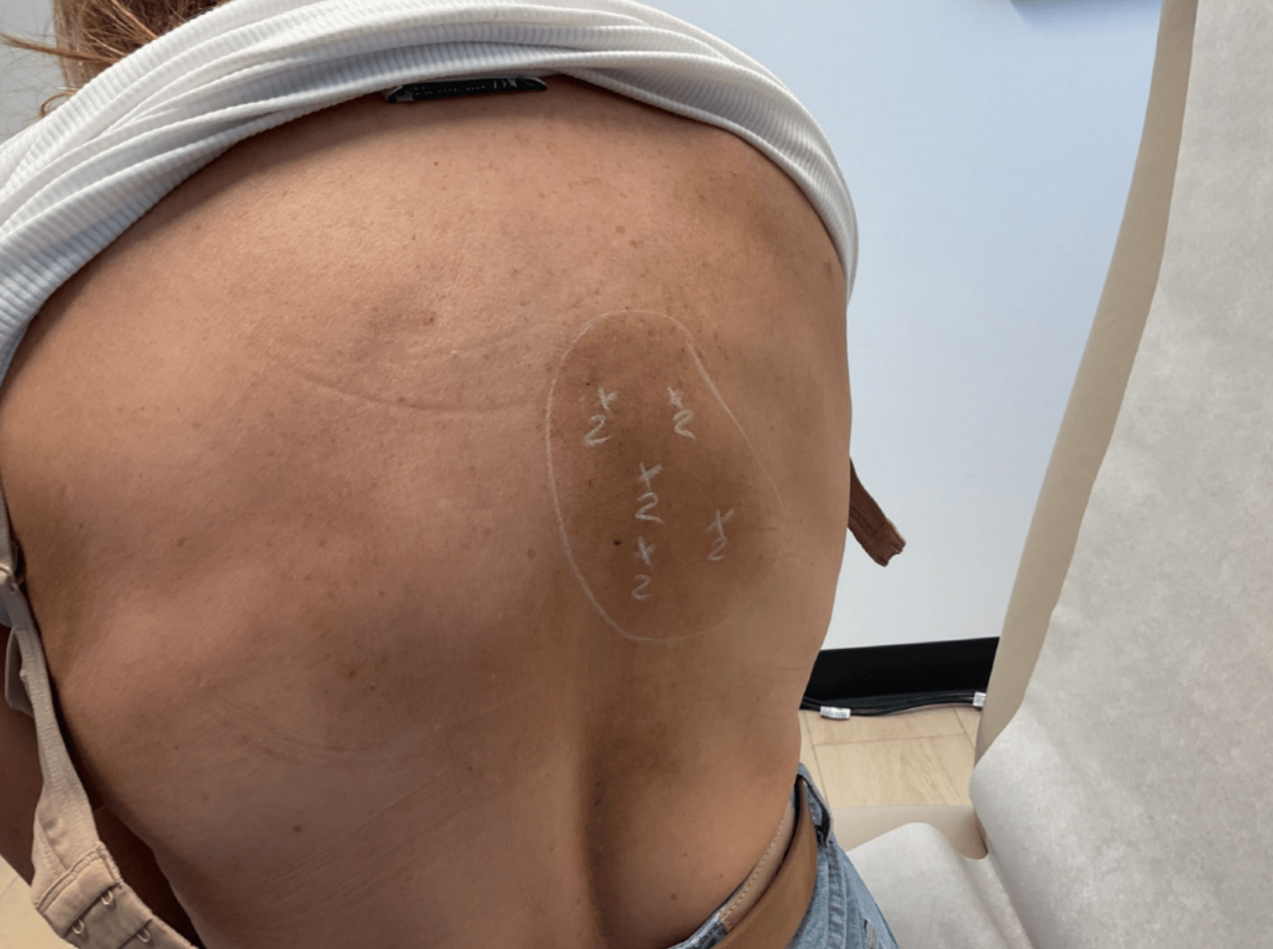
The patient's image during the treatment Intramuscular onabotulinumtoxinA injections (10 U total) were administered in a grid‑like pattern to the right upper back, targeting symptomatic dermatomes.

Given the refractory nature of her condition, the decision was made to trial botulinum toxin therapy. After discussing the risks, benefits, and off-label nature of the treatment, the patient consented to intramuscular injection of onabotulinumtoxinA (Botox). During the initial session, 10 units were administered to the right upper back in a grid-like pattern across the affected area (Figure 2). The procedure was well tolerated with no immediate complications.

Two weeks later, the patient returned for follow-up and reported partial improvement in symptoms but persistent pruritus in adjacent areas. A second therapeutic session was performed, during which a total of 26 units of onabotulinumtoxinA were injected: 18 units to the mid-back and eight units to the right upper back, again targeting the symptomatic dermatomes. The patient tolerated the procedure well and was provided with supportive educational materials about botulinum toxin therapy for neuropathic itch.

At the next follow-up visit, three weeks post-treatment, the patient reported substantial improvement in both intensity and frequency of pruritus, with her visual analogue scale (VAS) itch score decreasing from 8/10 at baseline to 2/10 at three weeks. She was able to resume her normal daily activities without significant discomfort and no longer required topical corticosteroids. No adverse effects or complications were noted throughout the treatment course. Overall, partial improvement was noted two weeks after the first injection, with marked symptomatic relief by the third week following the second session.

## Discussion

Notalgia paresthetica (NP) is a chronic cutaneous sensory neuropathy most commonly presenting as localized pruritus and dysesthesia along the medial scapular region, typically without an accompanying primary dermatologic lesion. It is thought to arise from entrapment or irritation of the posterior rami of the thoracic spinal nerves, most frequently T2 to T6, as they traverse the paraspinal musculature to innervate the skin. Contributing factors may include degenerative spinal changes, trauma, poor posture, or musculoskeletal imbalance [1,2]. Despite its prevalence, NP remains underdiagnosed and frequently mismanaged, leading many patients to suffer from prolonged, often debilitating symptoms.

Standard therapeutic options for NP include topical agents (e.g., corticosteroids, capsaicin), oral medications (e.g., gabapentin, amitriptyline), intralesional corticosteroids, physical therapy, and trigger point injections. However, these treatments often provide only partial or temporary relief, particularly in cases where the underlying neuropathic mechanism remains unaddressed [1,2]. Our patient’s course exemplifies this pattern: she failed both topical and intralesional corticosteroid therapy and experienced minimal symptomatic relief before transitioning to botulinum toxin therapy.

Botulinum toxin type A (BoNT-A) has demonstrated efficacy in treating various peripheral neuropathic pain syndromes, including trigeminal neuralgia, diabetic neuropathy, and postherpetic neuralgia [3-5]. Its mechanism of action in neuropathic pain involves inhibition of nociceptive neurotransmitters such as substance P, glutamate, and calcitonin gene-related peptide, leading to decreased peripheral sensitization and neurogenic inflammation. In addition, BoNT-A has been shown to downregulate pain-related ion channels and reduce ectopic neural discharges, which may further alleviate neuropathic symptoms [4,6].

The use of BoNT-A in NP is supported by a growing body of literature. A recent systematic review and meta-analysis evaluating BoNT-A for chronic peripheral neuropathic pain found statistically significant pain relief compared to placebo, with minimal adverse effects [3]. Reported adverse events in these analyses were generally mild and transient, including localized injection‑site discomfort and, rarely, transient muscle weakness, highlighting a favorable safety profile for BoNT‑A in neuropathic applications. Another meta-analytic study confirmed its efficacy in both muscle-based and non-muscle-based pain disorders, suggesting broad utility for chronic sensory neuropathies, such as NP [5]. Furthermore, case series and small clinical trials have demonstrated the effectiveness of intramuscular BoNT-A in reducing itch intensity and improving quality of life in patients with treatment-refractory NP [1,2]. Dosing regimens in the literature vary from 10 U to over 50 U, depending on the treatment area, with injections commonly distributed in a grid pattern 1-2 cm apart over the symptomatic dermatomes. This underscores the importance of individualized mapping to optimize response while minimizing dose exposure.

In the present case, the patient reported substantial improvement in pruritus after two sessions of targeted BoNT-A injections to the symptomatic dermatomes. The incremental dosing strategy allowed for both diagnostic assessment and therapeutic effect, ultimately providing long-awaited symptom control without adverse events. This case aligns with existing literature supporting BoNT-A as a safe and effective off-label treatment for NP, particularly when first-line therapies fail. Although our patient experienced substantial improvement, longer follow‑up is needed to determine the duration of benefit and whether maintenance injections are required. A broader case series would help validate optimal dosing intervals and long‑term efficacy.

## Conclusions

Notalgia paresthetica is a frequently underrecognized sensory neuropathy that can be challenging to treat, especially when conventional therapies such as topical corticosteroids and intralesional injections fail to provide sustained relief. The short follow‑up period in this case limits conclusions regarding the durability of symptom relief or maintenance dosing requirements. This case highlights the potential role of botulinum toxin type A as an effective and well-tolerated therapeutic option for patients with refractory NP. Given its ability to modulate peripheral neurotransmission and reduce neuropathic symptoms, botulinum toxin should be considered in select cases where standard interventions are unsuccessful. Further research is warranted to establish optimal dosing, injection techniques, and long-term outcomes in the treatment of NP with botulinum toxin.
